# From the evaluation of existing solutions to an all-inclusive package for biobanks

**DOI:** 10.1007/s12553-016-0175-x

**Published:** 2017-01-12

**Authors:** Heimo Müller, Nicolas Malservet, Philip Quinlan, Robert Reihs, Matthieu Penicaud, Antoine Chami, Kurt Zatloukal, George Dagher

**Affiliations:** 1 Medical University of Graz, 8010 Graz, Austria; 2 Institut national de la santé et de la recherche médicale, 75654 Paris, France; 3 School of Computer Science, Advanced Data Analysis Centre, University of Nottingham, Nottingham, NG8 1BB UK

**Keywords:** Biobanks, Software evaluation, Open source software

## Abstract

The domain of biobanking has gone through many stages and as a result there are a wide range of commercial and open source software solutions available. The utilization of these software tools requires different levels of domain and technical skills for installation, configuration and ultimate us of these biobank software tools. To compound this complexity the biobanking community are required to work together in order to share knowledge and jointly build solutions to underpin the research infrastructure. We have evaluated the available tools, described them in a catalogue (BiobankApps) and made a selection of tools available to biobanks in a reference toolbox (BIBBOX) that are use-case driven. In the BiobankApps tool catalogue, both commercial and open source software solutions related to the biobanking domain are included, classified and evaluated. The evaluation covers: 1) “user review” by an authenticated user 2) domain expert: quick analysis by BBMRI members and 3) domain expert: detailed analysis and test installation with real world data. The evaluation is paired with a survey across the more “advanced” (from a technology perspective) biobanks to investigate what tools are currently used and summarises known benefits/drawbacks of the respective packages. In the second step we recommend tools for specific use cases, and install, configure and connect these in the BIBBOX framework. This service also builds on the existing work in the United Kingdom in seeking to establish the motivations for different stakeholders to become involved and therefore assisting in prioritising the use-cases based on the level of need and support within the research community. All tools associated to a use-case are available as BIBBOX applications (technically this is achieved by docker containers), which are integrated in the BIBBOX framework with central identification and user management. In future work we plan to share the acquired knowledge with other networks, develop an Application Programmable Interface (API) for the exchange of metadata with other tool catalogues and work on an ontology for the evaluation of biobank software.

## Introduction

Modern biobanking is a relatively new concept that has evolved over the years to become an essential part of biomedical research. [1] Thousands of biobanks worldwide collect bio-specimens with clinical and research data from millions of individuals in different stages of their lives, before, during and after disease. All of this information is a great source of knowledge to support fundamental biomedical research and has the potential to dramatically contribute to the development of better predictive, preventive, personalized and participatory (P4) healthcare.

The biobanking landscape is evolving from insulated local biospecimen repositories to robust organizations providing services that cover a large part of the biomedical research cycle. High-throughput technologies are more accessible to research-biobanking and the number of biobanks providing services that require large storage capability and parallel data analysis is increasing. Due to the growing complexity of biobanking, a wide range of commercial and open source software solutions were developed in recent years. The tools are available in different development stages (from alpha version to production releases) and require different domain and technical skills for installation, configuration and use.

At this time the number of solutions dedicated to the biobanks is growing. There are many different software approaches available, e.g. solutions to manage a biobank in a similar manner as a laboratory with the help of a LIMS, solutions dedicated to study-based biobanks, solutions with modules or solutions with extensions to join other working areas (genomic, imaging, etc.) However, for the community of biobankers the main question is “Which is the “one” software tool I should use for my new biobank?”, or if they already work in a biobank “What is the solution to replace my existing homemade database, to work more efficiently?”

These questions imply many conclusions. First, biobankers do not have a clear vision about what is available for their new business. The second conclusion is that they have little time to search for and compare existing solutions. This second conclusion is backed up by the response received when suitable software is cited: *“How can I use that?”* or *“Do you have a demo somewhere?”*. Addressing the second demand is difficult compared to other scientific disciplines, as the biobank user community requires special assistance in basic Information Technology skill sets. Out of 10 biobanks, 8 declared no resources for developing IT projects (survey done in France 2014). Therefore we see a substantial need for connecting Biobanks with external informatics based experts.

A catalogue of software tools was an easy and pragmatic solution to help Biobanks. First, we made this list publicly available, and invited software providers to add their tools. In collaboration with the BBMRI-ERIC community, we setup evaluation mechanisms to share knowledge and improve the software selection process. In the next step we developed a demo and evaluation framework within the BBMRI-ERIC common service IT for well-defined scenarios using the BIBBOX framework.

## Related work

The NASA Software Catalogue provides an overview about general purpose scientific software packages [2] and the EGI Applications database (AppDB) [3] collects metadata about software tools integrated with the EGI infrastructure. Both catalogues cover a wide range of scientific disciplines.

In the life science and bioinformatics domain the European research infrastructure ELIXIR, provides the software tools platform https://bio.tools [4] covering both a tool registry and service registry. The European bioinformatics community generated a curated registry and the associated EDAM ontology [5] by running several community-driven hackathons and knowledge exchange workshops. The ELIXIR tools and data services registry evaluates bioinformatics methods in terms of quantitative performance and user friendliness. Further domain-specific catalogues of tools and web services are the BioCatalogue [6], BioDBCore [7] or myExperiment [8], just to mention some of the many catalogues and registries in the bioinformatics field.

Stol and Babar [9] compare different open source software evaluation methods and Taibi et al. developed an OpenBQR, a framework for the assessment of open source software tools [10, 11].

## BiobankApps

In the BiobankApps tool catalogue both commercial and open source software solutions are classified and tagged within the following categories:

- BIMS / LIMS / Sample Management;
- Data Integration / Data Warehouse / Cataloguing;
- Study Management, Phenotype Data Handling, EHR;
- Analytics and Visualization;
- Genotype / Omics Tools;
- Imaging;
- Data and Communication Middleware
- Helper Tools as transcoding, harmonisation and statistics.

**Fig. 1:**
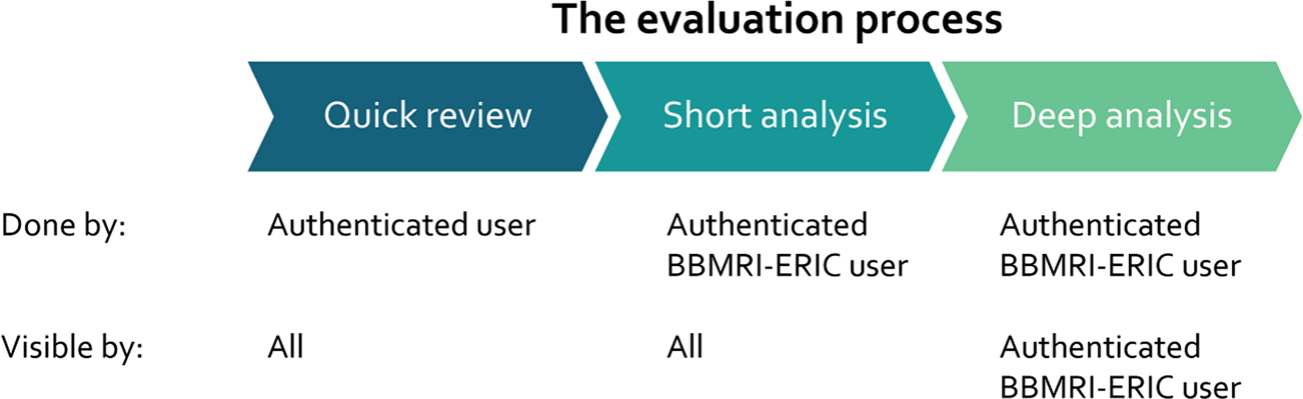
BiobankApps evaluation process

Related to this catalogue of tools, we built an evaluation process with different levels of information for the biobank community. The evaluation process consists of three steps, shown in Fig. 1:

1. Quick review: each authenticated user on the catalogue can add a rating
2. Domain expert quick analysis: BBMRI members add a short analysis in different categories and
3. User expert detailed analysis: The tool is installed and tested with real word data by BBMRI members.

The results of the short and deep evaluation stages are grouped by: domain oriented attributes, deployment and installation description, usability attributes and sustainability measurements. The evaluation is paired with a survey across the more “advanced” biobanks (from a technology perspective) to investigate what tools are currently used and the known benefits/drawbacks. Table 1 shows the evaluation questions of each analysis level, and the average time to address these questions.

**Table 1:** The BiobankApps evaluation process and timelines

Analysis level	Scope of questions	Evaluation time
User review	Rate with one to five stars	1 h
	Community driven	
	Allow comments	
Domain expert quick analysis	What are the goals of the tools?	4–8 h
	What are the main features?	
	How can it be helpful for biobanks?	
	What is the added value of the tool?	
Domain expert detailed analysis	More than 10 questions around the usability of the tool, the coverage area of the functionalities related to the biobanking activities etc.	More than 8 h.

**Fig. 2:**
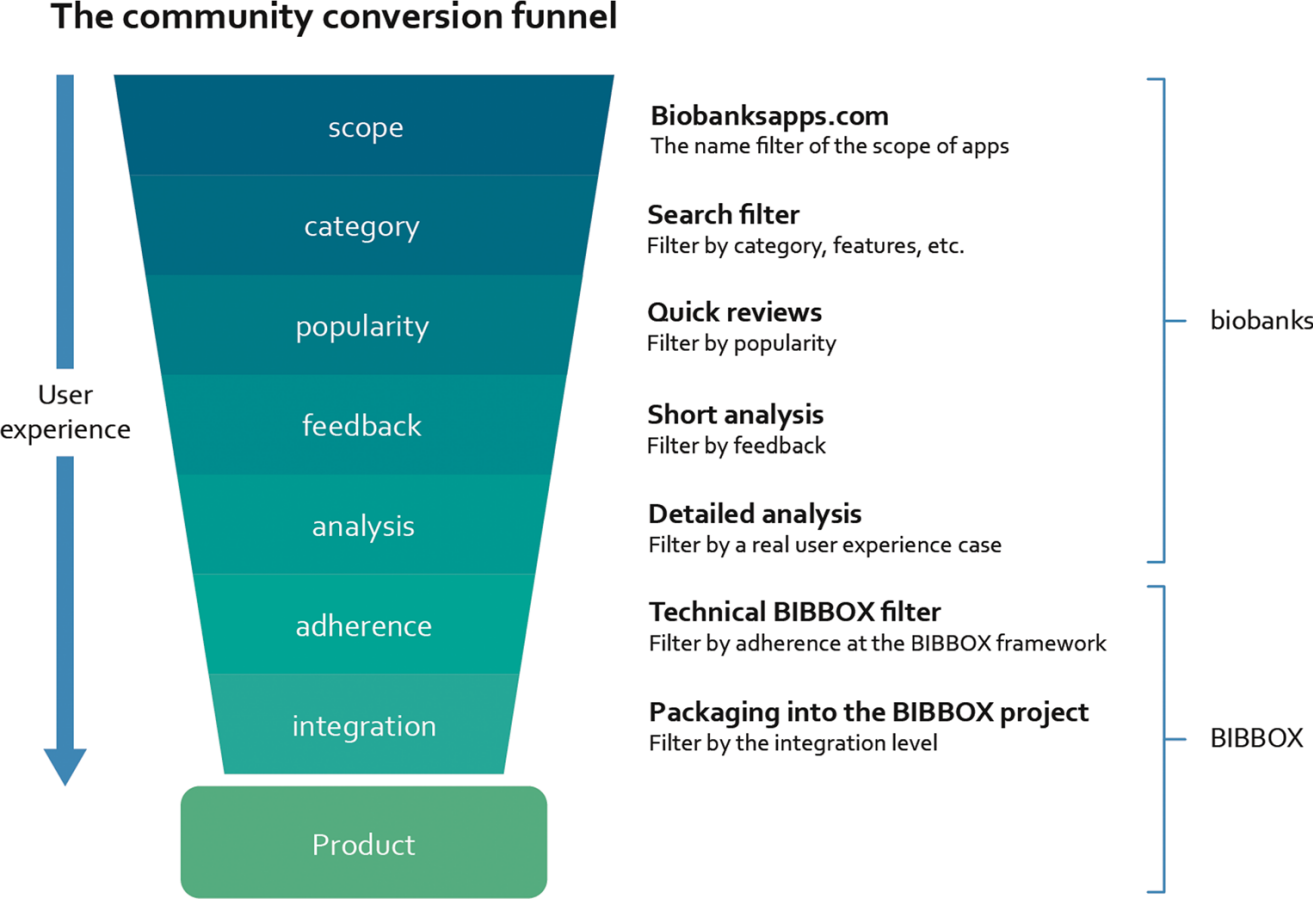
The schema of the community conversion funnel

Our deep analysis approach is based on the ISO/IEC 25010:2011 guidelines, see Fig. 2. These guidelines define characteristics to evaluate software in a standardized way.

## BIBBOX

Using the BiobankApps catalogue as starting point, we compiled tools for specific scenarios and installed, configured and connected these within a virtual machine. The definition of scenarios was done by a dedicated user requirement analysis provided by BBMRI-ERIC common service IT.

The scenario definitions build on the existing work of the BBMRI United Kingdom national node in seeking to establish the motivations for different stakeholders to become involved and therefore assisting in prioritising the use cases based on the level of need and support within the research community. The use cases will also extend to develop an understanding surrounding the capability of biobanks across Europe to fulfil such requirements. This insight is particularly useful for BBMRI-ERIC common IT services, as it seeks to identify the current gaps that need to be addressed before use cases can be successfully fulfilled. Examples of scenarios are a study-based DNA / liquid biobank; a clinical biobank focusing on cancer tissues and digital pathology or a collection of cell lines and plasma for a specific rare disease.

**Fig. 3:**
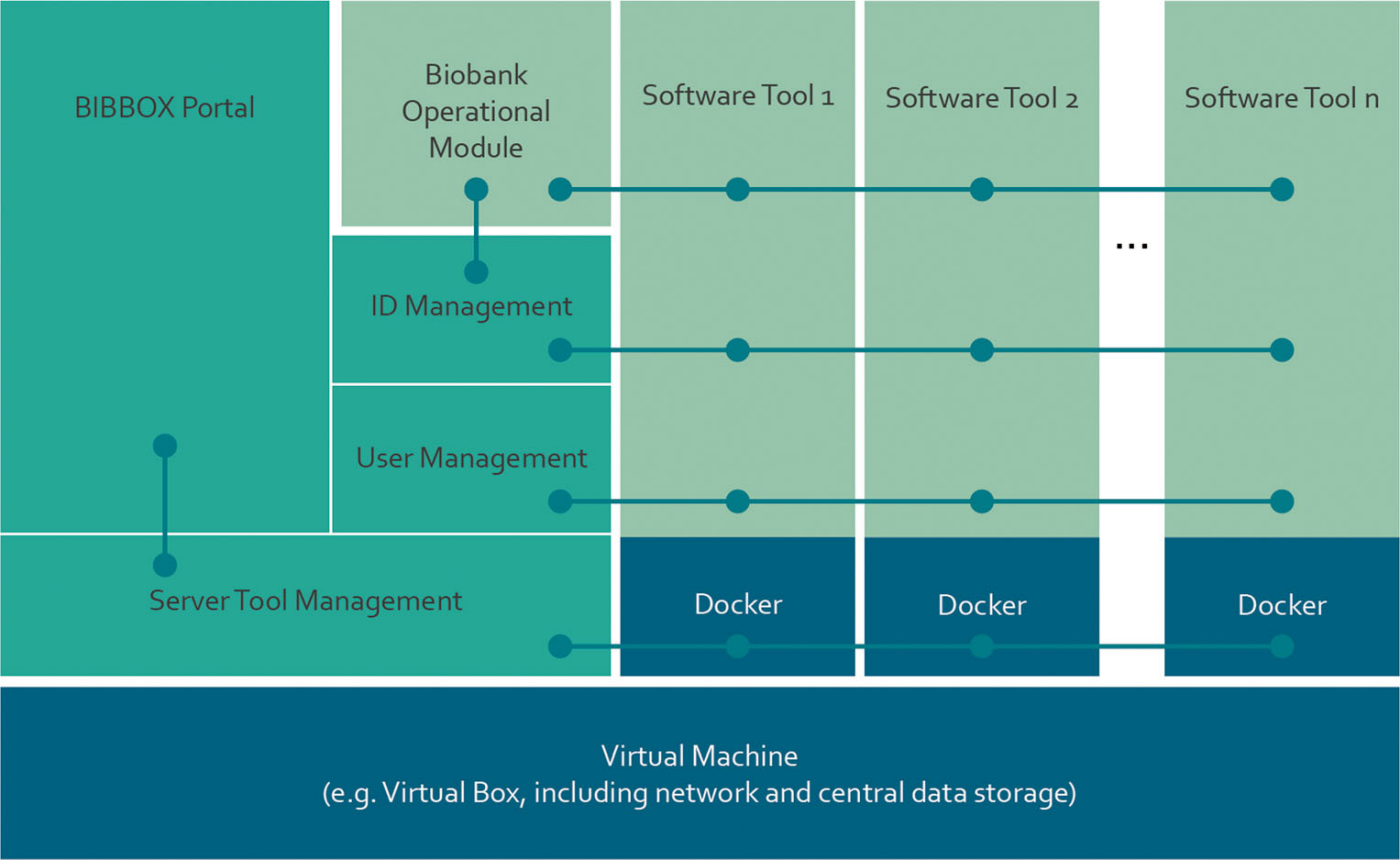
BIBBOX System Architecture

Tools necessary to cover the functionality of a scenario are selected from BiobankApps and “dockerized”, i.e. they are installed within docker containers, which can then be integrated and orchestrated in the BIBBOX (**B**asic **I**nfrastructure **B**uilding **BOX**) framework. For this task the BIBBOX framework provides functionality for the deployment of docker containers, a central ID and user management and a process monitoring dashboard, see Fig. 3.

In all scenarios the biobank operational module (BOM) covers the core functionality to operate a biobank, e.g. collection / study management, sample acquisition and sample metadata management, sample processing, sample storage, sample and data retrieval/distribution as well as data integration and cataloguing. With the help of an ID management system data objects describing samples, patients or medical records are linked between the biobank operational module and all other “dockerized” software tools. Each part of the biobank operational module is described with generic attributes, as defined by BiobankApps, and in addition by a functional classification as described in http://bibbox.org/biobank-operational-module. This list of functional requirements was generated on the basis of the ISBER Best Practices for Repositories, Collection, Storage, Retrieval, and Distribution of Biological Materials for Research by harmonizing several requirements and recommendation documents [12] and in addition gathering requirements through interviews with Biobank IT managers and IT representatives of BBMRI-ERIC national nodes. BBMRI.uk (also known as the UKCRC Tissue Directory and Coordination Centre) have been undertaking work to try and understand the various motivations, concerns and profiles of different stakeholders in biobanking. The development and refinement of personas allows the different groups to be represented in a manner that can be easily communicated (https://www.biobankinguk.org/personas/). These personas can be used in any engagement events to test if they do indeed accurately represent the different users and further work can be undertaken to determine if subgroups exist within each persona. These personas are then combined with different user stories and user flows to ensure the software and tools developed are tied to a specific user persona and identified use case.

**Fig. 4:**
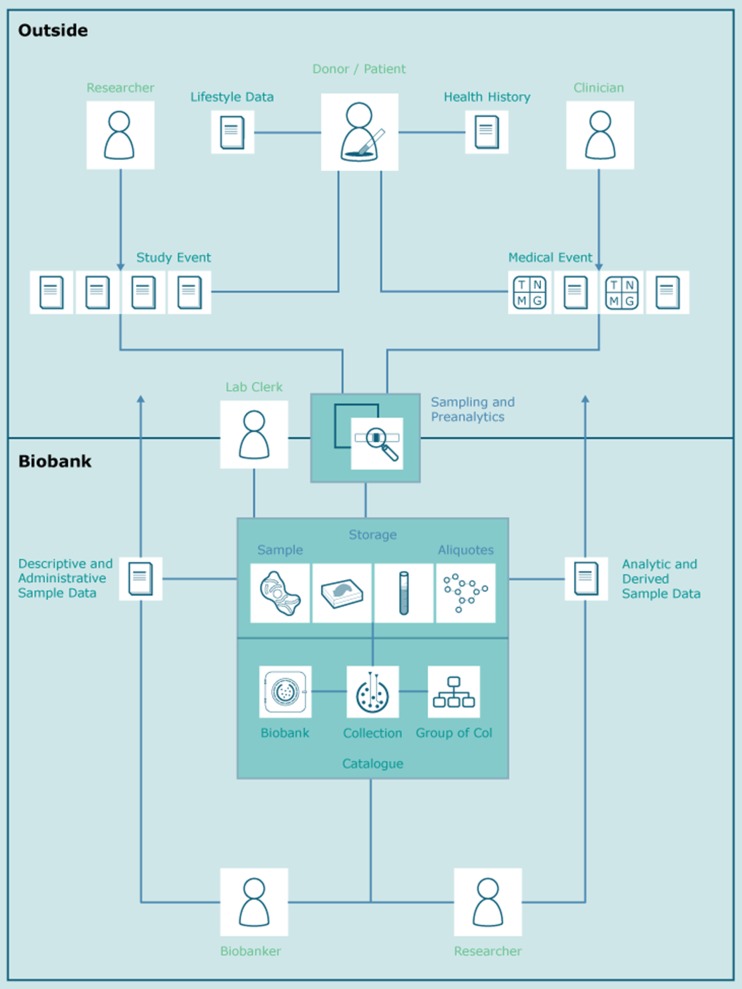
Stakeholders and data objects in a biobank environment

Data exchange between software tools and ID management follows the MIABIS recommendations [13]. MIABIS is the “de facto” biobank information standard for the BBMRI-ERIC community and has been widely accepted within Europe and beyond. Based in MIABIS we distinguish in our architecture between the following data objects, see Fig. 4:

- **Patients, Donors, Subjects or other direct person-related data.** This group includes data objects, which have a direct connection to a real person, e.g. demographic attributes, lifestyle data, or name and occupation of a doctor.
- **Medical or study event related data:** This group includes data objects, which describes a procedure or observation or were generated by a medical or study act. Provenance metadata of medical/study events consists of (a) information about a study and/or a medical case (b) additional patient/donor/subject data, and (c) information about the responsible doctor/scientist. The attributes of data objects should follow well-defined ontologies, e.g. an HL7 CDA or SNOMED for a diagnosis.
- **Sample, specimen or aliquot related data:** This group includes data objects, which are either used in the administration of a sample (location, storage temperature) or generated with specific analytical methods from a sample, e.g. laboratory values or NGS analysis results.

Each data object is named by an identifier (ID), which itself can be characterized with the following metadata attributes describing the

- **Scope of the identifier**
  - Global unique ID (**GUID**), e.g. the NIH Global Unique Identifiers [14]
  - Locally unique ID (**LUID**), either in a BIBBOX scope or locally within an organisation/biobank.
  - Tool specific ID (**TSID**) here the ID is only unique within a specific tool.
- **Identifiability of personally identifiable information (PII)**
  - fully identifiable (**FIID**) i.e. name/location/type of the object is embedded in the ID and/or in the denoted data object.
  - coded by organization ID (**COID**), i.e. all personal identifiable information was removed from the ID and denoted data object by the organization itself.
  - coded by trusted third party ID (**CTID**) i.e. all personal identifiable information was removed from the ID and denoted data object by a trusted third party, the organization itself cannot re-identify a person.
- **Persistence of the object denoted by the identifier**
  - ID denotes an object at a specific point in time, here the attributes of the object are immutable (**IMID**), e.g. an ID to a medical record or a diagnosis.
  - ID denotes an object, which can change over time, the attributes of the data object are dynamic (**DYID**), i.e. the ID of a sample administration record covering storage locations and aliquot availability.

For all software tools installed in a specific BIBBOX instance the identifier management component describes this meta information for the different data classes used and provides a graph database for provenance description of data objects and their causal dependencies based on the Open Provenance Model [15] and the W3C provenance data model [16].

Software tools to be included in the BIBBOX framework have to fulfil the following requirements, see Table 2.

**Table 2:** Prerequisites for software tools to be included in BIBBOX

	type	policy
listed in biobank.app	organizational	mandatory
open source / freemium license	organizational	mandatory
active user group and support	organizational	recommended
user/technical documentation / training	organizational	advantageous
available as *NIX web service	technical	mandatory
permission and role management	technical	mandatory
LDAP support	technical	recommended
SSO support	technical	recommended
data export/import functionality	technical	recommended
data access API	technical	advantageous
official docker container	technical	advantageous

## Future outlook

In the planning and setup of BiobankApps and the BIBBOX framework we faced technical challenges and had to decide on architectural issues and ontologies, but of equal importance we involved all stakeholders in the process and actively build a community. In the future we will further enhance the community building process by addressing the needs of different stakeholder groups (software developers, IT administrators and end users). In our community building strategy we will analyse the current needs and abilities of the community and understand what they care about. We will stimulate people to join, both for just visiting the tool catalogue and - most importantly - to actively contribute with their feedback, and we will connect the virtual catalogue to real-life events such as conferences and meetings.

The UK will be undertaking further work, both nationally and also across the BBMRI national nodes, to develop the understanding behind the use-cases, and the validation of the personas in order ensure that any services and software developed are in line with user expectations, and play to their motivations rather than their fears and concerns. As an example, there is a desire to explore the underlying motivation that may prevent the adoption of software tools. Although technical capabilities in biobanks are low, it cannot be expected that users will simply install and use tools once they become available. Ongoing evaluation and communication concerning the tools will be a continuing effort.

On the technical side we will investigate the Open Archives Initiative Protocol for Metadata Harvesting OAI-PMH, the W3C Data Catalogue Vocabulary (DCAT) and the FAIR data exchange principles as possible protocols and API for exchange and harvesting of metadata with other tool catalogues. In addition, we will work on a dedicated ontology for functional descriptions and evaluation of open source biobanks as well as commercial software.
